# Cardiac performance mirrors the passive thermal tolerance range in the oyster *Ostrea edulis*

**DOI:** 10.1242/jeb.249750

**Published:** 2025-02-04

**Authors:** Sandra Götze, Carl J. Reddin, Isabel Ketelsen, Michael Busack, Gisela Lannig, Christian Bock, Hans-O. Pörtner

**Affiliations:** ^1^ Alfred Wegener Institute, Helmholtz Center for Polar and Marine Research, Sections Integrative Ecophysiology and Deep-Sea Ecology & Technology, Am Handelshafen 12, 27515 Bremerhaven, Germany; ^2^ Museum für Naturkunde - Leibniz Institute for Evolution and Biodiversity Science, Invalidenstraße 43, 10115 Berlin, Germany

**Keywords:** Passive thermal capacity, OCLTT, Global warming, Bivalve, NMR spectrometry, Cardiomyocyte

## Abstract

Increasing frequencies of heatwaves threaten marine ectotherm species but not all alike. In exposed habitats, some species rely on a higher capacity for passive tolerance at higher temperatures, thereby extending time-dependent survival limits. Here, we assessed how the involvement of the cardiovascular system in extended tolerance at the margins of the thermal performance curve is dependent on warming rate. We studied organismal and heart tissue cellular responses of the European oyster, *Ostrea edulis*, challenged by rapid warming (+2°C per hour) and gradual warming (+2°C per 24 h). Starting at 22°C, cardiac activity was monitored as temperature was increased, tracking cardiac performance curves. Hearts were collected at discrete temperatures to determine cardiomyocyte metabolic profiles. Heart rate peaked at a lower Arrhenius breakpoint temperatures (ABT) of 30.5°C under rapid warming versus 33.9°C under gradual warming. However, oysters survived to higher temperatures under rapid than under gradual warming, with half of oysters dying (LT_50_) by 36.9°C versus 34.8°C, respectively. As rapid warming passed 30°C, heart rate fell and cardiomyocyte metabolic profiles suddenly changed as oysters switched to anaerobic metabolism for survival. By 36°C, severe fluctuations in Krebs cycle-related metabolites accompanied cardiac failure. In contrast, oysters exposed to gradual warming made gradual, extensive adjustments to intracellular metabolic pathways, prolonging aerobic cardiomyocyte metabolism to higher temperatures. This extended survival duration and ABT, beyond which cardiac activity decreased sharply and ceased. Our results emphasize how the rate of warming forces a trade-off between temperature maxima and survival duration, via tissue- and cellular-level impacts. European oysters possess adaptations that enable extended tolerance and survival of intertidal populations.

## Teaser

**Summary:** Whole-organism responses, cardiac activity and heart metabolites of oysters, *Ostrea edulis*, challenged by rapid and gradual warming beyond their thermal optimum range indicate that the total capacity of passive thermal tolerance is equal regardless of warming rate.

## INTRODUCTION

Human-induced global warming threatens marine ectotherms (Cabral et al., 2019; IPCC, 2022) as their performance depends fundamentally on ambient seawater temperatures (Pörtner, 2002; Hochachka and Somero, 2002; Kassahn et al., 2009), which fluctuate strongly during marine heatwaves. For long-term survival, organisms must cover their routine performance entirely via aerobic metabolism, which is the case within the optimum range of their species-specific thermal performance curve, between pejus limits, because only here do oxygen demand and supply to tissues fully support organism performance (the concept of oxygen and capacity limited thermal tolerance, OCLTT; Pörtner, 2010; Pörtner et al., 2017; Pörtner, 2021). Suboptimal temperatures cause a progressive mismatch in oxygen demand and supply and, when aerobic metabolism cannot be maintained, anaerobic energy supply may set in, prolonging survival but depleting energy reserves. Thermal stress due to suboptimal temperatures has a negative impact on growth, reproduction and fitness of ectotherms, eventually affecting entire populations, their interactions with other species and their distribution (e.g. Gazeau et al., 2014; Schalkhausser et al., 2014; Thomas and Bacher, 2018; Masanja et al., 2023). The progressive increase in global mean sea surface temperature (SST) by ∼0.7°C above preindustrial temperatures (see IPCC, 2019) is exacerbated by an increased frequency of heat waves, which substantially challenges species and ecosystems, causing latitudinal displacements and mass mortalities in coastal waters (Beck et al., 2011; Tan and Ransangan et al., 2019; Guo et al., 2022). However, species may display varying capacities in their tolerable heat load, often championed by the sessile marine ectotherms adapted to live in shallow water, especially intertidal habitats. Such capacities may be adjustable by acclimation (i.e. heat hardening; Georgoulis et al., 2022), which then extends the endurance duration of suboptimal conditions.

A primary mechanism for organisms to secure oxygen supply in the warmth, thereby extending the thermal range of aerobic metabolism beyond the active range, is maintaining cardiac activity (Brand and Roberts, 1973; Widdows, 1973; Marshall and McQuaid, 1993; Campbell et al., 2007), ventilation and/or oxygen transfer, as well as keeping oxygen demand as low as possible. Further cellular mechanisms of heat tolerance also contribute, such as anaerobic metabolism, metabolic depression, antioxidative defence and heat shock protection mechanisms (Pörtner, 2002, 2010; Pörtner and Knust, 2007; Pörtner et al., 2017). These imply the species transition into a passive range of thermal tolerance beyond pejus limits which can extend either the duration of tolerated change or the limiting temperature reached (shown by Peck et al., 2009, in Antarctic marine species; see Pörtner, 2010, for a graphical analysis), or both to an intermediate extent. In other words, the faster the temperature changes beyond pejus limits, the higher the maximum temperature that can be endured but, conversely, the shorter the total duration of survival. Accordingly, the balance between time extension or tolerated extreme is related to the rate of change. That is, the total capacity of passive tolerance is the same regardless of whether the rate of warming is rapid or more gradual, with a time component and a temperature component that both contribute to passive tolerance (see Pörtner, 2010). Differences in such passive capacity may partially underlie differential survival of populations and species in a warming world (Liao et al., 2021), such as those observed in the fossil record of hyperthermal events (Reddin et al., 2021), or in meta-analyses of climatic stress experienced by extant marine species (e.g. Reddin et al., 2020; Costello et al., 2023).

Here, we focused on the role of the cardiac system and its metabolic characteristics in the thermal tolerance of the European oyster, *Ostrea edulis*, extending our previous work on bivalves (Eymann et al., 2020; for *Mytilus edulis*, see Zittier et al., 2015). Linking cardiac tissue activity and cardiomyocyte cell metabolism hierarchically to individual survival, the rate of change, maximum endured temperature and time-limited tolerance may help describe the transition into the passive thermal range for the oyster. Early studies in molluscs relied on invasive techniques such as drilling a hole into the body interior to place electrodes into the pericardial cavity (e.g. Helm and Trueman, 1967; Feng and van Winkle, 1975). The considerable disruption of the organism made cardiac interpretation difficult (Veitch, 1974; Koester et al., 1979); hence, the development of a non-invasive method based on infrared-photo transducers by Depledge and Andersen (1990) was groundbreaking. Since then, cardiac activity has been extensively studied in marine invertebrates (e.g. Pandolfo et al., 2009; Bakhmet et al., 2012; Zittier et al., 2015; Xing et al., 2016, 2019; Gurr et al., 2018; Eymann et al., 2020). Cardiac activity can follow a bell-shaped, temperature-dependent performance curve, with low heart rates at both ends of the species’ thermal tolerance (e.g. Frederich and Pörtner, 2000; Xing et al., 2016; Eymann et al., 2020). The temperature at which cardiac activity is maximal and declines thereafter is commonly depicted (in ectotherms including fish) by the intersection of the increasing and decreasing regression lines, giving the Arrhenius breakpoint temperature (ABT; e.g. Lannig et al., 2004; Braby and Somero, 2006; Casselman et al., 2012; Hansen et al., 2017; Ghaffari et al., 2019; Dong et al., 2020).

Studies of cardiac cellular metabolism can detail mechanisms of limitation on cardiac activity. Studies of the cardiac metabolism of bivalves from the late 1960s to 1990s relied on measuring each metabolite of interest separately (e.g. Irisawa et al., 1969; de Zwaan and Wijsman, 1976; Collicutt and Hochachka, 1977). Modern techniques, such as mass spectrometry (MS) or nuclear magnetic resonance (NMR) spectroscopy, allow simultaneous screening and identification of numerous metabolites. When combined, these produce a species- and tissue-specific metabolic profile, which in itself is a sensitive indicator of environmental stress, toxicity and disease (e.g. Jones et al., 2008; Lannig et al., 2010; Ellis et al., 2014; Palma et al., 2019). Baseline concentrations of metabolites and their changes in response to environmental stress by MS or NMR spectroscopy have been investigated in bivalve gill, mantle or muscle tissues (e.g. Tuffnail et al., 2009; Tikunov et al., 2010; Cappello et al., 2018; Bock et al., 2024). However, few studies examined cardiomyocytes, the cells responsible for heart contraction (Hanana et al., 2012, 2014; Aude et al., 2019). Thus, little is known about how bivalve cardiac metabolism responds to climatic stress, or how metabolism relates to cardiac performance.

*Ostrea edulis* is an ecosystem engineer and marine bivalve species that, following historical overexploitation, is endangered in its natural habitat (Beck et al., 2011; Pogoda, 2019). Its order, *Ostreida*, has appeared relatively robust under rapid warming of both short-term experiments and ancient events of rapid global warming (Reddin et al., 2020, 2021). In a previous study, we defined the (acute) thermal window of *O. edulis* including its thermal optimum (18–24°C), the upper pejus temperature (*T*_pejus_, 24–26°C) and critical temperature (*T*_crit_, >26°C) along a warming gradient (2°C per 48 h) starting at 14°C until reaching upper thermal limits of around 36°C (Eymann et al., 2020). Building on this, we aimed to relate *O. edulis* survival and cardiac activity to the underlying metabolism of cardiomyocytes in a whole-organ context in response to warming. We targeted intermediates of the tricarboxylic acid (TCA, or Krebs) cycle. To account for the complex correlations in time-limited survival, we challenged specimens in two different warming regimes (2°C per hour and 2°C per 24 h), starting from the upper end of their thermal optimum range (22°C). These rates model extreme conditions potentially experienced by this species following tidal exposure or permanent submergence, respectively, during a heat wave. Temperatures were increased not until environmental maxima but until tolerance was exceeded, which allowed us to test whether individuals challenged at the faster rate achieved higher maximum temperatures and displayed shifts in aerobic versus anaerobic components of cardiac metabolism compared with those challenged by a longer, slow-rate warming. The results should deepen our understanding of how physiological processes shape the transition to and limit a bivalve's passive capacity in the species' pejus range.

## MATERIALS AND METHODS

### Animal origin and maintenance

Scuba divers of the Biological Station of Toralla (ECIMAT, University of Vigo, Spain) collected wild adult *Ostrea edulis* Linnaeus 1758 in the estuary of the Ría de Ferrol (Spain, 43°27.64′N and 8°12.04′W). Surface seawater temperatures were ∼13°C in October 2020 and ∼16°C in March 2021. Oysters were flown to the Alfred Wegener Institute, Helmholtz Center for Polar and Marine Research (AWI, Bremerhaven, Germany) and pre-acclimated to the institutional aquarium systems for at least 4 weeks (∼15°C and full salinity). During this time, oysters were fed 3 times a week with a mixed algal diet containing *Rhodomonas* sp. and *Paeodactylum tricornutum.* Prior to the experiments, oysters were brought to 20°C in two steps each overnight (first 18°C, then 20°C) and kept for ∼1 week. All specimens were cleaned of epibionts, measured and weighed. Oysters were of mixed sex and adult ages, weighing 95.3±31.8 g and measuring 7.4±0.8 cm in length and 7.3±1.0 cm in width (mean±s.d. in all cases). No ethical approval was needed for bivalves from local legislation or from the German Council for Animal Care for this study.

### Experimental setup

All experiments were conducted in recirculating experimental systems (total volume∼600 l, with a flow-through per aquarium of 200 ml min^−1^) starting at 22°C (upper thermal optimum range of European oysters collected from the Ría de Ferrol; Eymann et al., 2020) and ending when all oysters died. In the rapid warming protocol, the temperature increased progressively by 2°C per hour (continuous increase by 0.033°C min^−1^). In the gradual warming protocol, the temperature increased stepwise by 2°C every 24 h (increase accomplished within 2 h and temperature held constant for 22 h). Warming protocols approximate the exposure of the species to an intolerable heatwave when in the intertidal (rapid warming) or when submersed in shallow waters (gradual warming; discussed further towards the end of Discussion). Both ramps were carried out repeatedly, rapid warming *n*=9 and gradual warming *n*=6, each with multiple animals randomly drawn and reserved for assessing mortality, for tissue sampling and a small number for cardiac activity (details in sections below). Throughout all experiments, the water chemistry was closely controlled (Table S1). Temperature and the partial pressure of oxygen (*P*_O_2__) were monitored with a Fibox-3 device (PreSens) every 15–30 min independent of the warming rate. In the acute warming protocol, the partial pressure of carbon dioxide (*P*_CO_2__) and pH were measured immediately before and after the experiment and daily during the gradual warming protocol (Table S1). Carbon dioxide was measured using a Vaisala device (Vaisala, Finland). The pH was converted to freescale as recommended in the guide for best practice in ocean acidification research (Waters and Millero, 2013; Riebesell et al., 2011). Oysters were fed once a day with algal mixture to prevent starvation, which meant that oysters in the rapid warming exposure, which lasted less than 10 h, were fed prior to the start of exposure but not during the exposure. The gradual warming exposure lasted several days, so feeding once a day meant that these oysters were fed during exposure. Aquaria were cleaned regularly of faeces.

### Survival

The survival percentage was calculated separately for nine rapid warming ramps (2°C h^−1^), each starting with ∼12 oysters, and six gradual warming ramps (2°C 24 h^−1^), each starting with ∼22 oysters. Dead specimens, identified by their gills being clutched together and no longer distinguishable from each other (example photos in Fig. S1), were removed immediately from the aquaria. Rapid warming oysters were constantly watched for signs of death while the water parameters were monitored. The lethal temperature for half of the subjects (LT_50_) was determined using the online LT_50_ calculator by AAT Bioquest, Inc. (https://www.aatbio.com/tools/lc50-calculator). Differences between ramp LT_50_ values were tested using a *t*-test. The survival rate was plotted as mean±s.d. across replicate ramps with SigmaPlot 12 (Systat Software, Inc.).

### Cardiac activity

Cardiac activity was monitored using an improved non-invasive method (Eymann et al., 2020) based on the infrared photoplethysmography method developed by Depledge and Andersen (1990). Prior to the experiments, the shell area above the pericardium was sanded down carefully. An Optocoupler Vishay CNY-70 sensor was placed directly above the heart and glued to the shell with coral glue (EcoTech Elements). The signal was amplified with the Plethysmograph_E_Amplifier Rev.B02 (Dischereit, Aschberg, Germany). Specimens were submerged again after a few minutes and the glue hardened within 24 h. At this point, oysters were allowed to recover from possible handling stress for at least 1 week, when one oyster out of 20 died. Oysters were transferred to the experimental systems 48 h before exposures started. Plethysmographs were connected to a PowerLab (ADInstruments, Bella Vista, NSW, Australia) signal recording system and heartbeats were manually counted using LabChart (ADInstruments). Under rapid warming, counting always occurred during a 10 min interval after the desired temperature was reached. In case of signal disturbances, counting was shifted by a maximum of ±5 min, equating to a ±0.16°C temperature difference. During gradual warming exposure, the heartbeat was recorded throughout exposure to the respective temperature (for a maximum of 20 h as no recording took place during the 2 h feeding period), from which 10 intervals (picked by random draw throughout recording) of 10 min each were manually counted. Occasionally, the heart rate of single individuals could not be analysed at a particular temperature because of interfering noises. Based on the original heart rate data, various parameters were calculated (Table 1). The mean rate of increase of heartbeats per minute (beats min^−1^) with temperature was obtained from the linear slope of the straight line between the initial temperature and the temperature at maximum heart rate. ABT of both ramps were calculated in R (http://www.R-project.org/) as the intersections of the ascending and descending linear regressions of transformed variables (Nickerson et al., 1989; Yeager and Ultsch, 1989). The physiological thermal performance limit (temperature at which the heart rate is half-maximal; *T*_1/2*f*_H,max__) and the physiological thermal safety margin (pTSM) were calculated after Liao et al. (2021). pTSM was calculated as the difference between the maximum temperature oysters experience in the field (maximum August temperature over several years of 20.4°C reached in the Ria de Ferrol according to https://www.seatemperature.org/europe/spain/ferrol-august.htm) and the *T*_1/2*f*_H,max__ determined from our model. The average temperature at which the heart rate of oysters ceases (*T*_zero_), was calculated per warming rate as the lowest of two estimates: (1) the observed temperature when no individuals register a heart rate (*f*_H_) >0 beats min^−1^; (2) the temperature when the cardiac performance model (representing the population mean, see ‘Cardiac performance modelling’, below) decreases below 1 beat min^−1^. Eymann et al. (2020) narrowed the *T*_pejus_ for this population to between 22 and 26°C (marking the range between upper thermal pejus and *T*_crit_, respectively). Therefore, we consider 24°C as a reasonable estimate of *T*_pejus_ to calculate the exposure time (see Pörtner, 2010) until the LT_50_.

**Table 1.** Summary of heart and mortality associated parameters of *Ostrea edulis* exposed to either rapid (2°C h^−1^) or gradual (2°C 24 h^−1^) warming

	Temperature-dependent increase in *f*_H_ until ABT (beats min^−1^ °C^−1^)	ABT (°C)	LT_50_ (°C)	*T*_zero_ (°C)	*T*_1/2*f*_H,max__ (°C)	pTSM (°C)	Exposure time beyond *T*_pejus_ to LT_50_ (h)
Rapid	2.9±1.3	30.5	36.9±0.5	36.6	33.5	13.1	6.45
Gradual	3.4±0.7	33.9	34.8±0.2	36.0	36	15.6	129.6

The average increase per °C of heart rate (*f*_H_) was obtained from the linear equation between the initial temperature and the temperature of maximum *f*_H­_ per individual. The Arrhenius breakpoint temperature (ABT) was calculated according to Nickerson et al. (1989) and Yeager and Ultsch (1989) (Fig. 2). *T*_zero_ refers to the temperature at which *f*_H­_ dropped ultimately to zero. Temperature of half-maximal *f*_H_ (*T*_1/2*f*_H,max__) was derived from the cardiac model for the rapid (2°C h^−1^) exposure but the model for the gradual (2°C 24 h^−1^) exposure did not reach half of maximum (see Results). The physiological thermal safety margin (pTSM) was calculated from the difference between the maximal temperature oysters experience in the field (∼20.4°C in the Ria de Ferrol) and *T*_1/2*f*_H,max__. The exposure time from *T*_pejus_ (24°C) to half-maximal lethal temperatures (LT_50_) follows the model in Pörtner (2010) and quantifies the shift from maximizing the limiting temperature reached to maximizing the duration of tolerated change depending on the rate of warming. Variance estimates are shown ±s.d.

### Cardiac performance modelling

To estimate a mean cardiac thermal performance curve for Ría de Ferrol *O. edulis*, we used general additive mixed models (GAMMs) assuming a Poisson distribution (log link, fitted using penalized quasi-likelihood) and an autoregressive moving average (ARIMA) correlation structure to account for temporal dependence. The optimal correlation structure was assessed by comparing Bayesian information criterion (BIC) values of models with different ARIMA *p* and *q* values. Repeated measures of individual animals were modelled using random effects, with a random intercept for each individual. The smoothed additive term for temperature used *k*=7 for the rapid ramp and *k*=8 for the gradual ramp, representing the best balance between identifying the expected form of the curve (based on Eymann et al., 2020) and overfitting the data. GAMMs were fitted using R package mgcv (Wood, 2017) in R (http://www.R-project.org/).

### Heart metabolism

Tissues were sampled at 22, 26, 30 and 34°C (both exposures), and at 36 and 37°C (reached only in the rapid warming protocol). Specimens (rapid warming *n*=7–8 per temperature; gradual warming *n*=11–12 per temperature) were dissected on ice. Hearts (19.9±11.4 mg) were quickly snap-frozen in liquid nitrogen and stored at −80°C until further analysis. Soluble metabolites were extracted from the whole heart by methanol–chloroform extraction. Untargeted metabolic profiling was performed as described by Götze et al. (2020). Briefly, the metabolite-containing pellet was suspended in 55 µl deuterized water (D_2_O) comprising 3-(trimethylsilyl)-propionic-2,2,3,3-d4 acid, sodium salt as internal standard (TSP; 0.05 weight %; Sigma Aldrich, St Louis, MO, USA). One-dimensional ^1^H-NMR spectroscopy was carried out in an ultra-shielded vertical 9.4 T NMR spectrometer (Advance III HD 400 WB, Bruker-BioSpin GmbH, Ettlingen, Germany) equipped with a triple-tuned 1H-13C-15N-probe for 1.7 mm NMR tubes. Metabolic profiling was performed on spectra obtained with a Carr–Purcell–Meiboom–Gill (CPMG) sequence. All spectra were baseline, shim and phase corrected. Metabolites were identified using the software Chenomx NMR suite 8.1 (Chenomx Inc., Edmonton, AB, Canada) and calibrated to the TSP signal and specific dilution factor of each sample (55 µl D_2_O/fresh weight of the heart). NMR spectroscopy has a lower sensitivity than MS techniques, such that profiles of 30–40 metabolites are typical (see also Götze et al., 2020; Bock et al., 2024).

Statistical analysis for this section and pathway analyses used Metaboanalyst (Metaboanalyst 5.0; Xia and Wishart, 2016; Chong et al., 2019). Data were log transformed to obtain a normal distribution. Statistical analysis comprised unsupervised principal component analysis (PCA) to detect and remove outliers and a one-way ANOVA followed by a Tukey *post hoc* test. In-depth analysis of metabolic clusters used a supervised partial least-square discriminant analysis (PLS-DA) accounting for the weighted sum of squares of the PLS loadings (variable importance in projection, VIP score). Overfitting and importance were tested by permutation tests with a significance of class separation tested by 1000 permutations. Significant differences in individual metabolite levels were checked by an analysis of microarray (SAM), which also estimated the false discovery rate (FDR).

The settings for the pathway analysis were as described in Götze et al. (2020). Briefly, metabolites of the 22°C group (as the starting control) were compared against one of the higher temperatures, e.g. 30°C, within the same ramp, or we compared rapid heating versus gradual heating at the same temperature. Pathway analysis settings were as follows: ‘global test’ as enrichment method, ‘relative-betweenness centrality’ for topology analysis, and the pathway library of zebrafish, *Danio rerio*, as putative reference metabolome. Implausible pathways (e.g. pathways associated with plant or bacterial metabolism), highly dubious pathways (>30 compounds with one metabolite assigned) and pathway changes with a negative common logarithm of the *P*-value [−log10(*p*)] of less than 2.5 were excluded. Metabolite datasets were tested for statistical outliers via the Nalimov test, which were then excluded.

Amino acids that feature importantly in anaplerosis or cataplerosis in the TCA cycle were summed into groups according to their common precursors of pyruvate, oxaloacetate, succinyl-CoA, acetyl-CoA and fumarate, following table 1 in Owen et al. (2002). In summary, amino acids convertible into pyruvate comprise alanine, glycine, threonine and tryptophan. Those convertible into oxaloacetate comprise aspartate and asparagine. Those convertible into fumarate comprise phenylalanine and tyrosine. Those convertible into succinyl-CoA comprise isoleucine and valine. And those convertible into acetyl-CoA comprise leucine, isoleucine, lysine, phenylalanine, tyrosine, tryptophan and threonine. Their summed concentration change with temperature and differences between rates of warming were tested using linear regression. Medians were compared using Wilcoxon tests at the common temperature of 34°C. All tests were two-tailed.

## RESULTS

### Survival and LT_50_

**Fig. 1.**
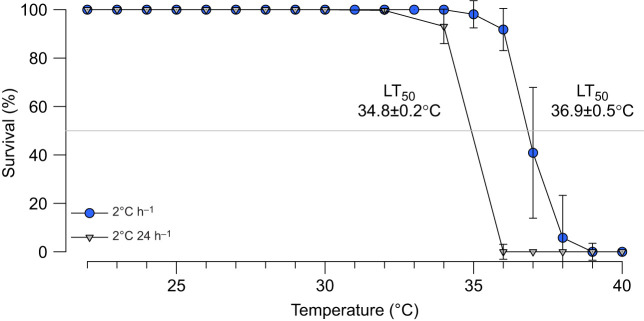
**Survival of *Ostrea edulis* subjected to rapid (2°C h^−1^) or gradual (2°C** **24 h**^−1^**) warming.** Survival was calculated for each replicate ramp separately (rapid warming: *n*=9 replicate ramps; gradual warming: *n*=6 replicate ramps) and expressed as percentage survival, starting from 12 oysters as 100% for 2°C h^−1^ and from 22 oysters as 100% for 2°C 24 h^−1^. The percentage of surviving oysters is shown for every 1°C temperature increase. The grey line indicates the level at which 50% of oysters remained alive (LT_50_) and the respective calculated LT_50_ values are shown.

Under rapid warming (2°C h^−1^; Fig. 1), the largest fraction of specimens died between 36°C and 37°C (from 91.8±8.7% to 40.9±27% alive). At 38°C, only 5.8±17.5% of oysters remained alive, and these died in the transition to 39°C. Oysters exposed to gradual warming (2°C 24 h^−1^) all survived until 32°C, beyond which mortality set in, except for one, which died within the first 12 h at 36°C, while the final oyster reached 38°C. Thus, calculated LT_50_ values were significantly higher for oysters under rapid warming, at 36.9±0.5°C (9 replicate ramps), than for those under gradual warming, at 34.8±0.2°C (6 replicate ramps; *t*-test *P*<0.001).

### Cardiac activity and associated indices

**Fig. 2.**
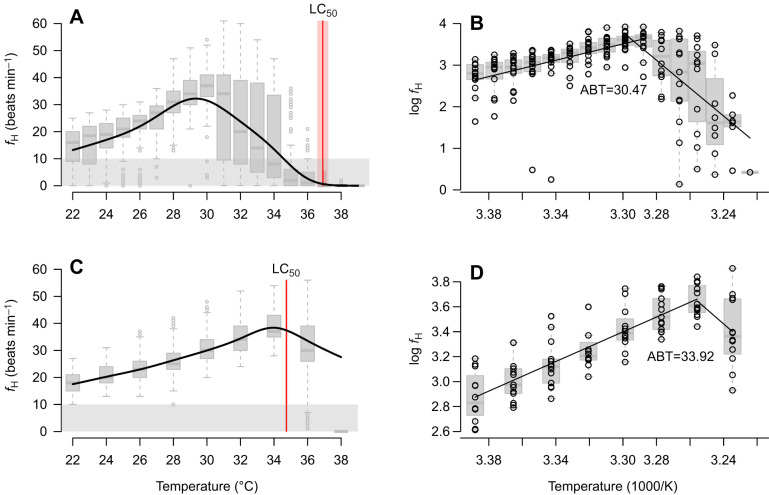
**Mean cardiac activity and Arrhenius plots of *O. edulis* subjected to rapid (2°C h^−1^) or gradual (2°C 24 h^−1^) warming.** The rapid warming exposure (A,B) comprises heart rate (*f*_H_) data of 9–16 oysters each counted over a 10 min interval per temperature step. The gradual warming exposure (C,D) comprises data of 10 times a 10 min interval for each of 12–13 oysters counted per temperature. Individuals with a *f*_H_ of <10 beats min^−1^ were classed as ‘inactive’, including individuals that died by this point (i.e. ‘died’ entries had a *f*_H_ of ‘0’, while entry failures are simply missing values. Box plots show the median (bold line) and interquartile range (IQR; box); whiskers cover observations within 1.5× IQR. In A and C, the general additive mixed model (GAMM) is a parametric model; details are presented in Table S2, estimating the conditional mean (curved line) rather than the medians of the box plots. In C, all values were zero (i.e. variance=0) by 38°C despite one oyster still living, so this step was modelled by extrapolating the GAMM above 36°C. In B and D, the Arrhenius breakpoint temperature (ABT) was the intersection of the two shown regressions for each rate of warming.

Oyster cardiac activity increased steadily from 14±7 and 18±4 beats min^−1^ at 22°C for rapid and gradual warming, respectively (*n*=15 and *n*=12; Fig. 2; Table S2), by about 3 and 3.4 beats min^−1^ °C^−1^ (Table 1) to maxima of 37±8 beats min^−1^ at 30°C and 39±2 beats min^−1^ at 34°C. The rapid warming post-maximum decline in cardiac rate was highly variable, with, for example, 10 out of 14 oysters maintaining high cardiac activity (≥30 beats min^−1^) at 31°C, while the remaining four oysters had no or few heartbeats (≤15 beats min^−1^). At 35°C, only two oysters were observed with heart activity between 20 and 30 beats min^−1^. All other surviving oysters (*n*=13) showed lower cardiac activity (<10 beats min^−1^), resulting in an average *f*_H_ of 7±10 beats min^−1^. At 36°C, cardiac activity was minimal (3±4 beats min^−1^). In contrast, under gradual warming, post-maximum cardiac rates remained similar to each other at 32±3 beats min^−1^ within the first ∼12 h at 36°C, but this suddenly declined to zero (*T*_zero_) within the last 10 h of exposure to 36°C, with only one oyster surviving until 38°C.

Thus, the cardiac ABT was lower for oysters under rapid warming, at 30.5°C versus 33.9°C for those under gradual warming (Fig. 2B,D). However, the very rapid decline in gradual warming heart rate resulted in a poorly defined downward slope (Fig. 2C,D) that complicated calculation, and therefore warming rate comparison, of *T*_1/2*f*_H,max__ and *T*_zero_ (Table 1)_._ Calculated physiological thermal safety margins differed between oysters exposed to different heating ramps, with a pTSM of 13.1°C during rapid warming and 15.6°C during gradual warming.

### Heart metabolism

**Fig. 3.**
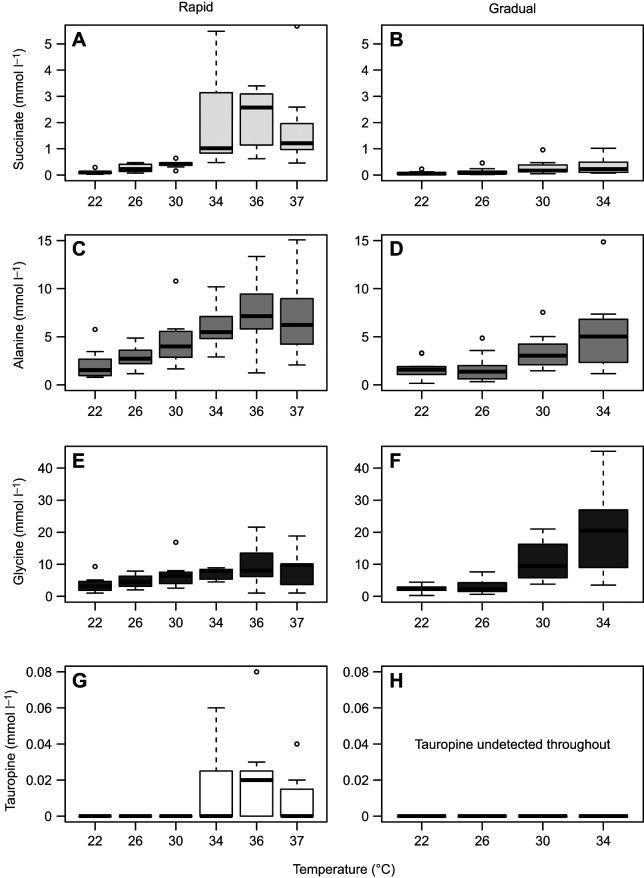
**The individual concentration changes of metabolic intermediates in *O. edulis* subjected to rapid (2°C h^−1^) or gradual (2°C** **24 h^−1^) warming.** Changes in succinate (A,B), alanine (C,D), glycine (E,F) and tauropine (G,H) following rapid (left) and gradual (right) warming exposure. Note how much higher concentrations of succinate are produced with rapid warming while gradual warming results in much higher glycine concentrations. Box plots show the median (bold line) and IQR (box); whiskers cover observations within 1.5× IQR. Rapid warming: *n*=8 until 26°C and *n*=7 thereafter; gradual warming: *n*=12 except for 26°C, which has *n*=11.

**Fig. 4.**
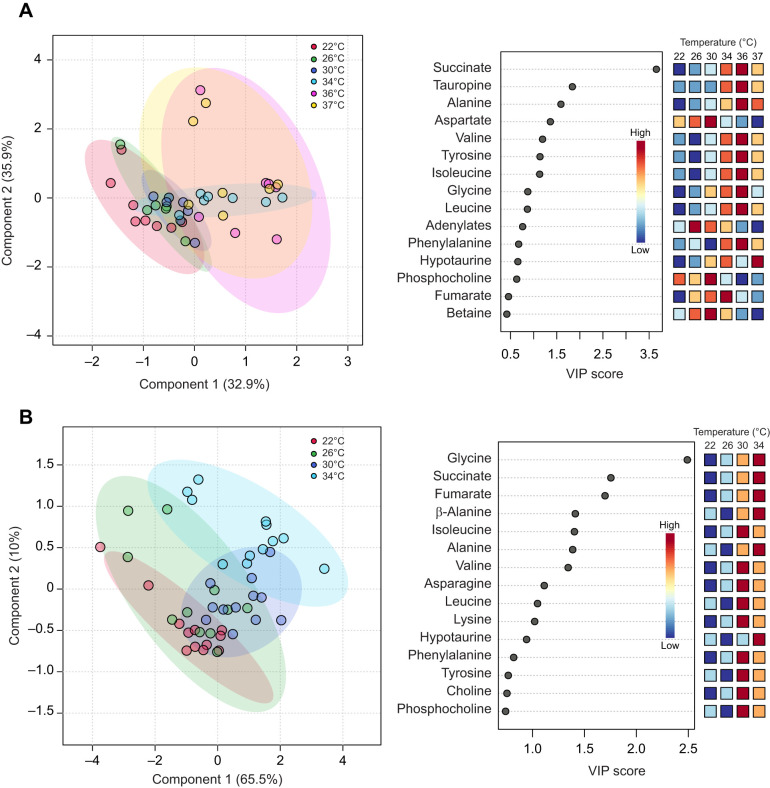
**The influence of warming rate on heart tissue metabolic profile of *O. edulis* challenged by either rapid (2°C h^−1^) or gradual (2°C** **24 h^−1^) warming.** Score plots (left) and variable importance in projection (VIP, right) scores of the partial least-square discriminant analysis (PLS-DA) model of assigned cardiomyocyte metabolites are shown for the rapid (A) and gradual (B) warming exposure. The ellipses in the score plots correspond to a 95% confidence interval and the coloured circles indicate individual samples at the respective temperature. The VIP score shows the weighted sum of squares of the PLS loadings. The colour-coded boxes next to the VIP scores reflect the concentrations of the respective metabolite per temperature step. Rapid warming: *n*=7–8 per temperature; gradual warming: *n*=11–12 per temperature.

We identified 32 metabolites in heart tissue, mainly osmolytes, amino acids and their derivatives (Table S3). While rapid warming caused significant changes in the concentration of six metabolites, 12 metabolites changed significantly with gradual warming, with only two shared, succinate and alanine, which peaked at the highest achieved temperatures of each warming ramp (Fig. 3). Rapid warming metabolic clusters were similar with low cluster variance until 30°C, when aspartate peaked and shortly after adenylates peaked (Fig. 4A; overall PLS-DA model *P*<0.001 on 1000 permutations). Thereafter, between 34°C and 37°C, adenylates crashed and metabolic profile ellipses deviated from the rapid warming lower temperatures (Fig. 4A), indicating a relatively sudden temperature-driven shift in heart metabolic equilibria. For instance, tauropine as the end product of anaerobic glycolysis was absent between 22°C and 30°C but significantly increased to become detected in about half of the samples taken at 34°C, 36°C and 37°C, in contrast to it not changing throughout in oysters under gradual warming (Table S3).

**Fig. 5.**
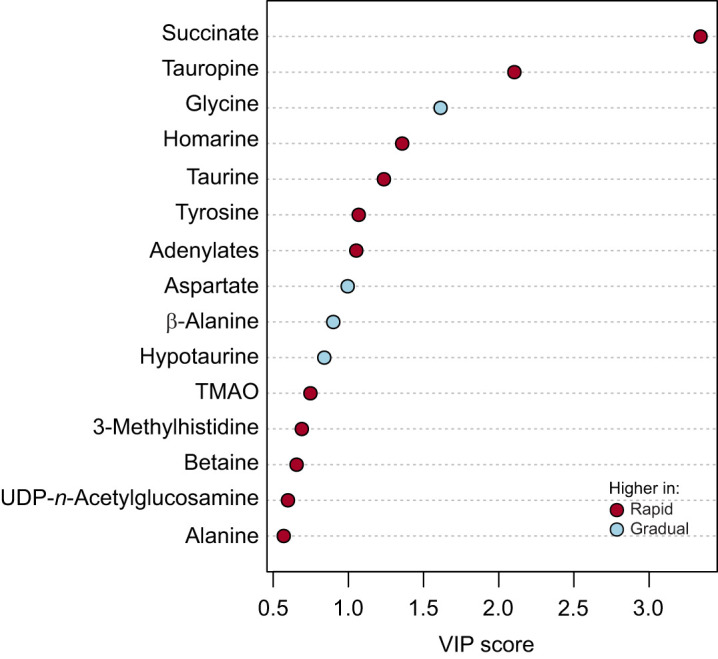
**Heart metabolites contributing most to the differences between the metabolic profiles following rapid and gradual warming at 34°C, the highest common temperature, as identified by PLS-DA VIP scores.** The point colours indicate whether relative concentrations of the corresponding metabolite are higher in the rapid warming or in the gradual warming condition, emphasizing a significant shift to succinate and tauropine during rapid warming (supporting statistical test results in Tables S3 and S4A).

Metabolic clusters separated less abruptly under gradual warming compared with rapid warming (Fig. 4B; left; PLS-DA model *P*<0.001 on 1000 permutations), shown by partially overlapping clusters, except for the earliest and latest clusters of 22°C and 34°C, as metabolites including glycine, succinate and fumarate gradually increased (VIP scores, Fig. 4B, right). Levels of isoleucine, leucine, lysine, valine, phenylalanine, threonine and *n*-acetylglucosamine were highest at 30°C under gradual warming, while alanine, β-alanine, glycine, succinate and hypotaurine levels peaked at 34°C (Table S3). In comparison, glycine levels were not significantly different between any steps of rapid warming (Table S3; Fig. 3). Despite peaking at 34°C, succinate remained significantly lower than in oysters under rapid warming (Figs 3 and 5; Table S4A). Significant changes specific to rapid warming included aspartate, tauropine, tyrosine and adenylates, comprising the sum of ATP, ADP and AMP (all *P*≤0.01, one-way ANOVA; Table S3), whereas those specific to gradual warming included β-alanine, phenylalanine, lysine, glycine, threonine, leucine, valine, isoleucine, UDP-*n*-acetylglucosamine and hypotaurine (all *P*≤0.004, Table S3).

### Cardiac metabolic pathways

Out of 33 putative metabolic pathways, the same 20 met the selection criteria; as for the individual metabolites, the short duration of rapid warming stress allowed fewer significant changes in pathways than gradual warming, changing five versus 14, respectively (Table S4B versus C). Under both rates, no pathways changed between 22°C and 26°C, but warming thereafter elevated levels of succinate, potentially feeding back on propanoate metabolism, butanoate metabolism and the TCA cycle. In addition to these three pathways, rapid warming to 34°C altered the metabolism of the amino acids alanine, aspartate, glutamate and tyrosine. In addition to those altered in rapid warming, gradual warming altered a broader suite of intracellular metabolic pathways, affecting the metabolism of additional amino acids, lipids, cofactors and vitamins, and aminoacyl-tRNA biosynthesis was detected.

### Cardiac catabolism of amino acids

Warming beyond the optimum (above 22°C) significantly increased four out of five of the TCA cycle intermediate groupings of amino acids, but only two out of five differed with the rate of warming (Fig. S2, Table S5). Those convertible to pyruvate increased significantly more strongly per °C under gradual warming, while those convertible to oxaloacetate stayed steady with temperature under gradual warming but significantly decreased with temperature under rapid warming. At the highest common temperature of 34°C, the only significant difference between ramps was that the fast ramp reached a higher median concentration for amino acids convertible to fumarate (*W*=59, *P*=0.01, rapid *n*=7, gradual *n*=12), which could push succinate formation. At 36°C and 37°C under rapid warming, all summed concentrations showed high variation between specimens (Fig. S2).

## DISCUSSION

Rapid climate change is increasingly challenging marine fauna, in both wild and aquaculture settings. Fundamental understanding of climate sensitivity and its underpinning mechanisms requires integration of all climate drivers in the ocean and a unifying concept across species, such as OCLTT. Such a concept allows assessment of species' climate resilience by linking the fundamental to realized niche and bridging to ecosystem-level consequences (Pörtner, 2021). Climate resilience can be assessed as the capacity of mechanisms shaping passive tolerance, which take over once the (pejus) limits of active tolerance, supported by an entirely aerobic metabolism, are surpassed. According to present findings, the consecutive range of passive tolerance plays out differently depending on the rate of warming (see Introduction; Pörtner, 2010), an observation that may reconcile apparently contrasting results from different experimental protocols addressing thermal tolerance. The surpassing of passive tolerance mechanisms is visible in current biogeographical shifts seen under climate change (IPCC, 2022), the net effect being habitat loss in warming seas (Liao et al., 2021). Beyond limited plasticity of thermal tolerance, evolutionary shifts in thermal limits may require far longer to become effective (Saupe et al., 2014).

As expected, *O. edulis* individuals challenged with rapid warming achieved higher temperatures (e.g. LT_50_) at the expense of exceeding aerobic cardiac metabolism and transitioning to anaerobic metabolism. This may have enabled survival but ultimately limited the period of survival at the highest temperatures, with death associated with cardiac failure. In contrast, individuals challenged with gradual, longer-term warming had more time for metabolic adjustments, resulting in less disturbance to aerobic cardiac metabolism at high temperatures, supporting a higher cardiac ABT and *T*_1/2*f*_H,max__ and survival for a longer duration, but reaching lower maximal temperatures (e.g. LT_50_). Loss of organism-level performance is often earliest and strongest, manifesting here as cardiac capacity limitations. Loss in organism performance (e.g. heart rate) in turn exacerbates performance loss at the tissue and cellular level (e.g. TCA cycle balance), which then further increases thermal sensitivity (Pörtner, 2002). It is key to identify how the responses of different tissues/organs contribute to whole-organism limits, considering the differences in their specific cellular homeostasis and metabolism (e.g. Tikunov et al., 2014; Bock et al., 2024). Assessment of functional and metabolic constraints at the organism, tissue and cellular level at thermal extremes thus supports an integrated evaluation of climate change effects on marine species, including coverage of potential heat wave conditions, with and without superimposed tidal cycling.

### Preadaptation to thermal environment

The loss of performance at the whole-organism level reflects the earliest level of thermal stress (Pörtner, 2002, 2010), linked to a drop in cardiac performance, itself through cellular metabolic constraints in the heart (see ‘Cellular responses scale up to extended survival’, below). The performance curve mirrors the fundamental ecological niche, whose characteristics are then also indicated by cardiac activity and thresholds such as cardiac breakpoint temperatures (ABTs; Somero, 2005). In marine bivalves, cardiac activity differs among species depending on lifestyle and their typical environmental temperatures, which vary with geographic distribution and depth (e.g. Xing et al., 2016; Ghaffari et al., 2019; Dong et al., 2022). For example, hearts of warm-adapted scallops such as *Argopecten ventriculosus* beat faster than hearts of cold-adapted scallops such as *Pecten yessoensis* (maximum 73.6±0.5 versus 31.5±0.5 beats min^−1^, respectively; Xing et al., 2016). Warm-adapted scallops are also able to maintain their cardiac activity at higher temperatures than cold-adapted scallops (ABT at 34.1±0.2 versus 22.0±0.2°C, respectively, corresponding to a difference of 20° in latitude; Xing et al., 2016). Similar heart characteristics related to thermal adaptation were reported for two intertidal oyster species, *Magallana gigas* and *Magallana angulata* (Ghaffari et al., 2019). The heart rate ABT was lower in *M. gigas* (∼55 beats min^−1^, 28.9±0.3°C) sampled from 10° higher latitude compared with *M. angulata* (∼60 beats min^−1^, 31.4±0.2°C). The relationship between temperature and heartbeat frequency may reflect decreasing pumping efficiency and increasing energy expenditure in warmer temperatures, due to the rise in resting metabolic rate (according to *Q*_10_). To compensate for reduced pumping efficiency and to meet increased energy demands, marine organisms adapt to warm habitats via higher pumping frequencies and increased capacities of cardio-ventilatory performance.

Despite historical evidence of oyster reefs in the lower intertidal (Kregting et al., 2020) and their local intertidal aquaculture (Montes et al., 1991), *O. edulis* lives predominantly submerged, affording less exposure to high temperature fluctuations. Adjustments to thrive in more thermally stable, submerged habitats may be reflected in heart characteristics, including lower maximum cardiac rates compared with those of more intertidal species. Maximum *f*_H_ of between 37 and 40.6 beats min^−1^ here, depending on the rate of warming, was lower than those of the more commonly intertidal *Magallana* species (∼60 beats min^−1^) (Ghaffari et al., 2019) or the mid-intertidal mussel *M. edulis* (∼45 beats min^−1^) (warming by 3°C per night; Zittier et al., 2015). Differences in habitat temperature variability may thus play a role in setting characteristics such as heart rate.

Our results show that the rate of warming did not affect the maximum cardiac activity close to the ABTs, which were similar in oysters of both experiments. However, initiation of cardiac arrhythmia shows that animals are clearly under stress before cardiac ABT (Eymann et al., 2020). During rapid warming, maximal cardiac activity of oysters occurred at around 30°C followed by a slow and variable decline in heart activity, as oysters survived warming by another 9°C past the ABT. Natural rhythms in behaviour, such as diel filtration rhythms, and their differential persistence over the short duration of rapid warming probably added to response variation between individual oysters (Fabra et al., 2025). In contrast, maximal cardiac activity of oysters exposed to gradual warming was shifted higher by 4°C but with a rapid collapse beyond the abt. This indicates that passive tolerance becomes exhausted over the longer duration of suboptimal exposure. Less reliance on anaerobic metabolism as an important element of passive tolerance parallels the faster collapse beyond the higher ABT of gradual warming (see below).

Despite a rather low pumping capacity, which resembles those of more cold-adapted bivalves, *O. edulis* displayed an impressive capacity to maintain overall cardiac activity under warming. Even when challenged with rapid warming of 2°C h^−1^, cardiac activity was upheld to 30°C, or to temperatures as high as 34°C and a higher ABT when warming was more gradual. Rapid warming (2°C h^−1^) led to higher rates of anaerobic contributions and higher maximal heartbeat frequencies at 30°C than in oysters exposed to gradual warming to the same temperature (37±8 beats min^−1^ compared with 31±3 beats min^−1^, respectively). The upward shift in ABTs during gradual warming may thus have been supported by some metabolic depression, resulting in the higher ABT at the same cardiac frequency. Interestingly, the shift in ABT (and thus organismal performance) seems to be related to improved cardiomyocyte oxygen metabolism, extended to higher temperatures (see below).

The remarkable heat tolerance of this marine bivalve species is possibly related to an evolutionary history of intertidal adaptation. For instance, intertidal bivalve body temperatures can reach over 40°C when exposed to solar radiation during low tide (genus *Mytilus;* Helmuth, 1998; Helmuth et al., 2006), and the flatline temperature *T*_flat_ (=*T*_zero_ in the present study) of the intertidal mussel *Mytilus californianus* occurred at 41.0±1.4°C (Moyen et al., 2022), about 5°C above the *T*_zero_ of *O. edulis*. As *T*_zero_ is found close to the lethal temperature (Table 1), this higher lethal threshold of the mussel may also relate to the higher warming rate, 9°C h^−1^, applied in Moyen et al. (2022)*.*

### Cellular responses scale up to extended survival

We identified tissue intermediates of carbohydrate and amino acid metabolism in hearts of *O. edulis* (de Zwaan and Wijsman, 1976; Collicutt and Hochachka, 1977). Warming by 2°C per hour appeared to be too rapid for major metabolic adjustments, resulting in a relatively sudden, temperature-driven shift in heart metabolic equilibria and finally a collapse in adenylates (Fig. S3) and, possibly, associated energy levels. Only 6 out of 32 metabolites, only those involved in pathways of carbohydrate and limited amino acid metabolism, changed in concentration throughout the exposure. In contrast, warming by 2°C per 24 h resulted in more gradual, but expansive and possibly regulated adjustments of cardiomyocyte metabolism. These included concentration changes of more numerous metabolites, demonstrating additional, more diverse metabolic pathways, including metabolism of lipids, cofactors and vitamins, and genetic information processing. Glycine, a glycogenic and cytoprotective intermediate, increased the most among intermediates identified under gradual warming (from 2.3±1.1 mmol l^−1^ at 22°C and 11.0±6.2 mmol l^−1^ at 30°C, to 20.7±13.0 mmol l^−1^ at 34°C; Fig. 3). Elevated levels of glycine are probably related to increased protein degradation and were found in king scallops (*Pecten maximus;* Götze et al., 2020) and abalone (*Haliotis fulgens*; Tripp-Valdez et al., 2017) after thermal stress alone or in combination with hypoxia and/or hypercapnia.

To better understand the metabolic constraints on bivalve cardiomyocytes, we analysed amino acids that can enter the TCA cycle for gluconeogenesis and lipogenesis. Overall, amino acids convertible to pyruvate increased more strongly per °C under gradual warming, possibly supporting a higher flux through the TCA cycle. Amino acids convertible to oxaloacetate remained virtually unchanged under gradual warming, which appears beneficial compared with rapid warming where they decreased significantly with increasing temperature, indicating a decline in aerobic metabolic contributions. In conclusion, the stabilization and support of aerobic metabolism may have supported the upward shift in ABT and extended survival during gradual warming. These conclusions are in line with the general notion that the tight regulation of TCA cycle flux is crucial for aerobic tissue functioning and is supported by anaplerosis (a process replenishing the pool of intermediates) and cataplerosis (which removes intermediates for other purposes) (e.g. Owen et al., 2002; Inigo et al., 2021). In humans, cardiac failure is associated with an imbalance between anaplerosis and TCA cycle flux (Des Rosiers et al., 2011; Nickel et al., 2013; Inigo et al., 2021).

In oysters exposed to rapid warming, summed levels of TCA-relevant amino acids remained unchanged for as long as cardiac activity was not yet maximal, indicating a balanced TCA cycle flux. However, at temperatures when cardiac activity declined under rapid warming, after reaching the ABT of 30.5°C, concentrations of intermediates were highly variable between oysters sampled in the same temperature group, indicating a correlated onset of TCA imbalance and cardiac failure.

Rapid warming also caused cardiomyocytes to resort to anaerobic metabolism more and thereby compensate to some extent for shortages in aerobic energy production. This involved metabolizing glycogen (carbohydrate metabolism) and aspartate (amino acid metabolism) to alanine and succinate, respectively, as well as glycogen to tauropine, as reflected in metabolite changes (significantly elevated succinate and tauropine concentrations) under rapid warming (Fig. 3) (for mitochondrial anaerobiosis in oysters, see de Zwaan and Wijsman, 1976; Collicutt and Hochachka, 1977; Ortmann and Grieshaber, 2003). The shift to anaerobiosis with its limited ATP yield indicates energetic constraints during thermal extremes (Pörtner et al., 2017). The onset of cardiomyocyte anaerobiosis occurred temporally sooner and probably at a lower temperature under rapid warming, indicated by the accumulation of fumarate from 26°C (Fig. 4) prior to increased levels of succinate beyond 30°C (Fig. 3; Tables S3 and S4). Moreover, succinate levels were twice as high in cardiomyocytes of oysters exposed to rapid warming versus gradual warming (0.4±0.2 versus 0.2±0.1 mmol l^−1^, respectively; *P*<0.05), despite similar initial values at 22°C (0.1±0.04 and 0.05±0.02 mmol l^−1^, respectively; *P*=0.51). This reflects the rapid onset of the glycolytic response and mitochondrial oxygen deficiency during rapid warming but also the capacity to acclimate at slower rates of warming, which, depending on the rate of warming, are both useful adaptations in intertidal environments. Under gradual warming, fumarate and succinate levels increased simultaneously from 30°C. Changes in metabolites were similar in *O. edulis* gill tissue under intermediate warming rates of 2°C per 48 h, showing that anaerobiosis was initiated beyond 26°C in the gill, at a somewhat lower temperature than in the heart (Eymann et al., 2020).

In line with the reasoning above, the increasing mismatch between cardiac oxygen consumption and supply caused progressive metabolic constraints and a reliance on anaerobic metabolism under rapid warming. The use of anaerobic metabolism may have supported an upward shift of heat death of the oysters to 37°C. Meanwhile, under gradual warming, summed levels of TCA-relevant amino acids were only slightly increased at 30°C, indicating that TCA cycle flux was still balanced despite small but observable mismatches between aerobic energy demand and supply. At 30°C, metabolic depression may have occurred and reduced oxygen demand, so that the heart had to beat less than during rapid warming (see responses to thermal regime in ‘Preadaptation to thermal environment’, above). Such depression may have contributed to the upward shift in cardiac ABT from 30.5°C under rapid warming to 33.9°C under gradual warming. However, as warming progressed to 34°C, metabolic adjustments became insufficient to maintain performance, with persisting oxygen deficiency causing organismal performance to rapidly decline.

The Spanish oyster population that we investigated, despite living submerged, possesses a remarkable heat tolerance beyond their thermal optimum, in line with a capacity of this species for life in the intertidal. Before overexploitation, this population probably also occupied intertidal zones and traditional *O. edulis* farming in the regional intertidal continues (Montes et al., 1991). Heart rate and associated metabolic responses of European oysters to rapid and gradual warming indicate that the Spanish population of *O. edulis* lives far from their lethal thermal limits while permanently submerged. Seasonal seawater fluctuations, kept cool by coastal upwelling (Chabrerie and Arenas, 2024), can be expected between 11 and 21°C (August mean 18.8°C, NW of Spain in Galicia, www.seatemperature.org). However, mean seawater temperature may increase by 2.6°C (1.6–3.5°C as 5–95% range among CMIP5 models for RCP8.5) by the end of the century, with 10 times as many heat wave days compared with the recent past (IPCC, 2019, 2022). For instance, Bernal-Ibáñez et al. (2023) analysed Bay of Biscay marine temperature records to inform their choice of experimental control and heatwave seawater temperatures of 20°C and 24°C, respectively, and 28°C heatwaves under RCP8.5 projections. They also found median heatwave durations of 7.5 days. For mechanistic completeness, our experiments pushed the organisms past recorded environmental temperatures, as they may be pre-adapted to conditions not experienced, or difficult to record, in current, submersed populations used in the present study. If the current trajectory of global warming is maintained, safety margins (pTSM) for intertidal aquacultures towards the warm edge of this species' range may be removed, progressively eliminating aquaculture viability. Synergistic stresses including hypercapnia and hypoxia will also diminish the pTSM of *O. edulis* and cause intermittent stress for local submersed oyster populations. Widespread warming, ocean hypoxia and acidification are not unique to modern times and probably raised extinction rates during ancient hyperthermal events (Kiessling et al., 2024). Although not all oyster species may be as well adapted to warming stress as *O. edulis*, *Ostreida* in general may be more robust than many other marine ectotherm clades (Reddin et al., 2021). Adaptability to intertidal life, especially increased capacity for passive tolerance, is likely to be a phylogenetically conserved characteristic that additionally enhances survival during climate warming, especially if thermally buffered retreats are available.

### Conclusions

We assessed how underlying metabolic processes relate to organismal performance in cardiac tissue of the oyster *O. edulis*. Our findings deepen the understanding of how the cardiovascular system supports the organism's passive tolerance (e.g. via anaerobic metabolism and, possibly, metabolic depression) when challenged by suboptimal temperatures. Our results emphasize that the rate of warming has a significant role in how survival is extended, cardiac activity is sustained and cardiomyocyte metabolism is stabilized, such that differences in warming rate may explain why different experiments, with all else being similar, obtain disparate results. While the total capacity of passive tolerance is supposed to be the same during rapid versus gradual warming, there is a time component and a temperature component that both contribute to passive tolerance to variable degrees (see Pörtner, 2010). The time component of the passive range may be extended during gradual warming by adjustments for a larger contribution of aerobic metabolism through stabilization of the TCA cycle, and an associated shift of cardiac breakpoint to higher temperatures. This may also be supported by metabolic depression, while the use of anaerobic energy production is more limited compared with rapid warming. During more rapid warming and the associated shortening of exposure periods at any temperature (Peck et al., 2009), the capacity for passive tolerance is probably similar (see Pörtner, 2010) but relies on tolerance of higher temperature at the expense of survival time. Anaerobic mechanisms may then have a stronger role in taking oysters to higher maximal temperatures (higher LT_50_) compared with gradual warming (36.9 versus 34.8°C, respectively).

Rapid warming saw a slow and variable decline in heart activity beyond ABT of cardiac activity, enabling survival of another 9°C warming past the breakpoint temperature. The 4°C upward shift of cardiac ABT during gradual warming was followed by a rapid collapse beyond the breakpoint temperature and a lower maximum temperature of survival, indicating a trade-off between components of thermal tolerance. It reflects that the time component of passive tolerance can only be extended at the expense of the temperature component and vice versa. The mechanisms behind this trade-off remain obscure, especially in terms of what is causing the exhaustion of passive tolerance over the duration of suboptimal exposure. For example, the question arises whether the limited use of anaerobic metabolism is a primary cause or a trade-off with metabolic depression? This relative metabolic depression extended survival and the fractional contribution of aerobic metabolism, but was not enough to keep lethal thermal limits (LT_50_) at the same higher temperatures as seen during rapid warming. Overall, intertidal adaptations probably make this species relatively robust to climate change within *Ostreida* and exemplify the increased robustness to climate change of *Ostreida* relative to other marine ectotherm clades. Our study also shows how maintained cardiac performance and resilience play a key role in setting passive thermal tolerance and in shaping whole-organism resilience to thermal stress.

## Supplementary Material

10.1242/jexbio.249750_sup1 Supplementary information
