# Supplementary material for: Cardiac performance mirrors the passive thermal tolerance range in the oyster Ostrea edulis
Source: J Exp Biol. 2025 Feb 4;228(2):JEB249750. doi: 10.1242/jeb.249750 (PMC11832122; doi:10.1242/jeb.249750)
Supplement: Supplementary information [file jexbio-228-249750-s1.pdf]

(A)

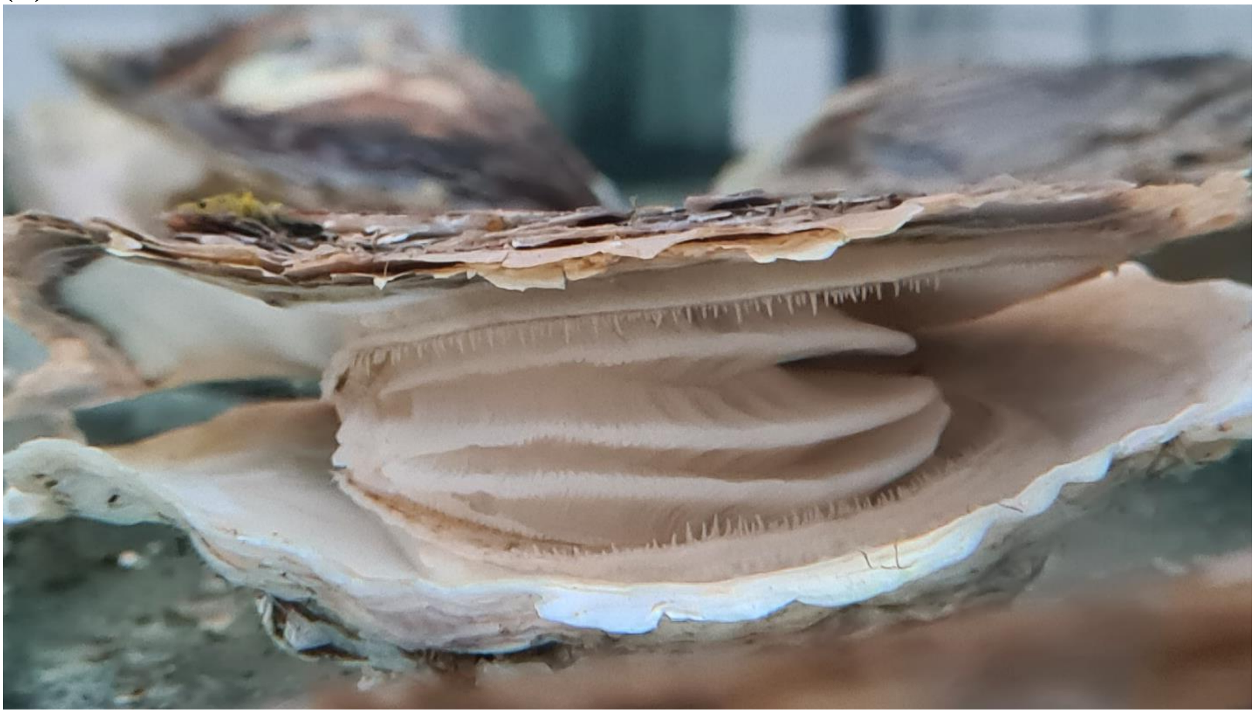

(B)

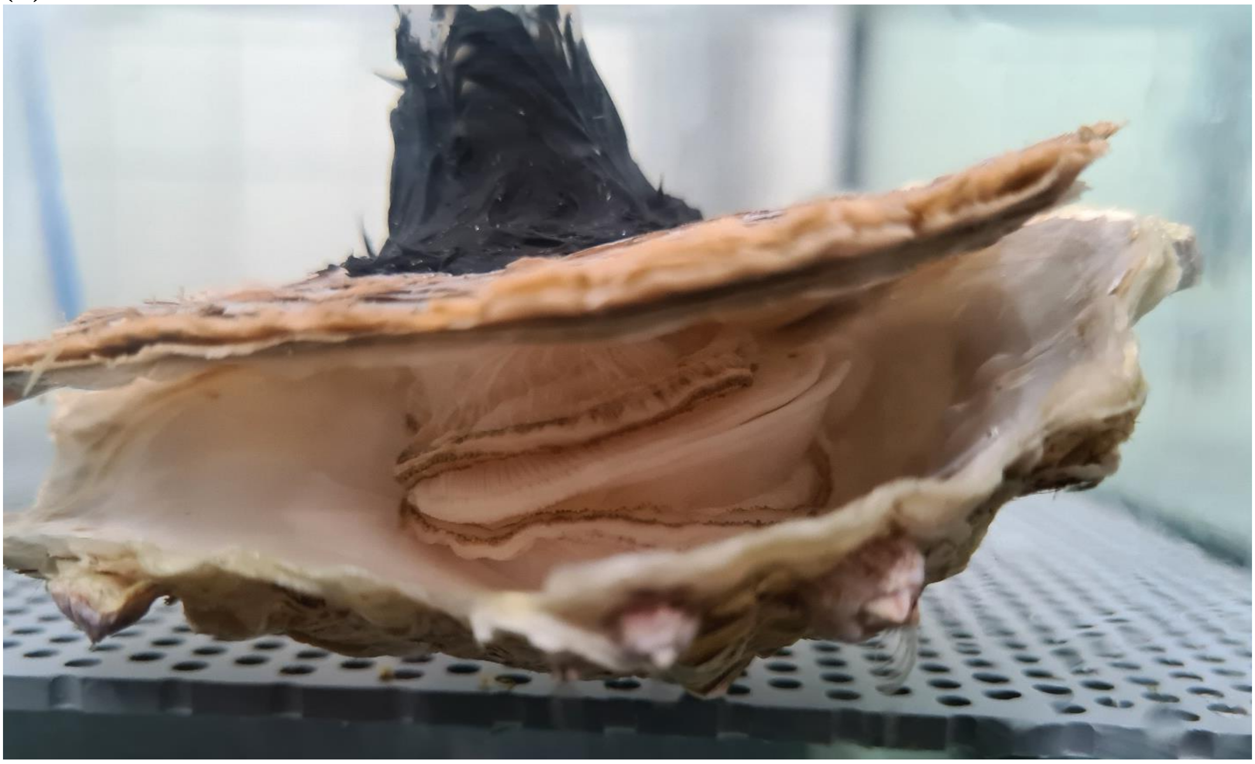

Fig. S1. Example photographs showing gill appearance in (A) alive oysters, showing separated, functional gills (centre), and (B) dead oysters, with no separation of gills. After a certain temperature the oysters remained open, though alive (also observed and mentioned in Eymann & Goetze et al. 2020), so our main criteria (besides oysters with plethysmographs attached, as in (B), showing zero signal) became that live oysters had their gills spread filtering water, while dead oysters' gills were clutched together and no longer distinguishable from each other.

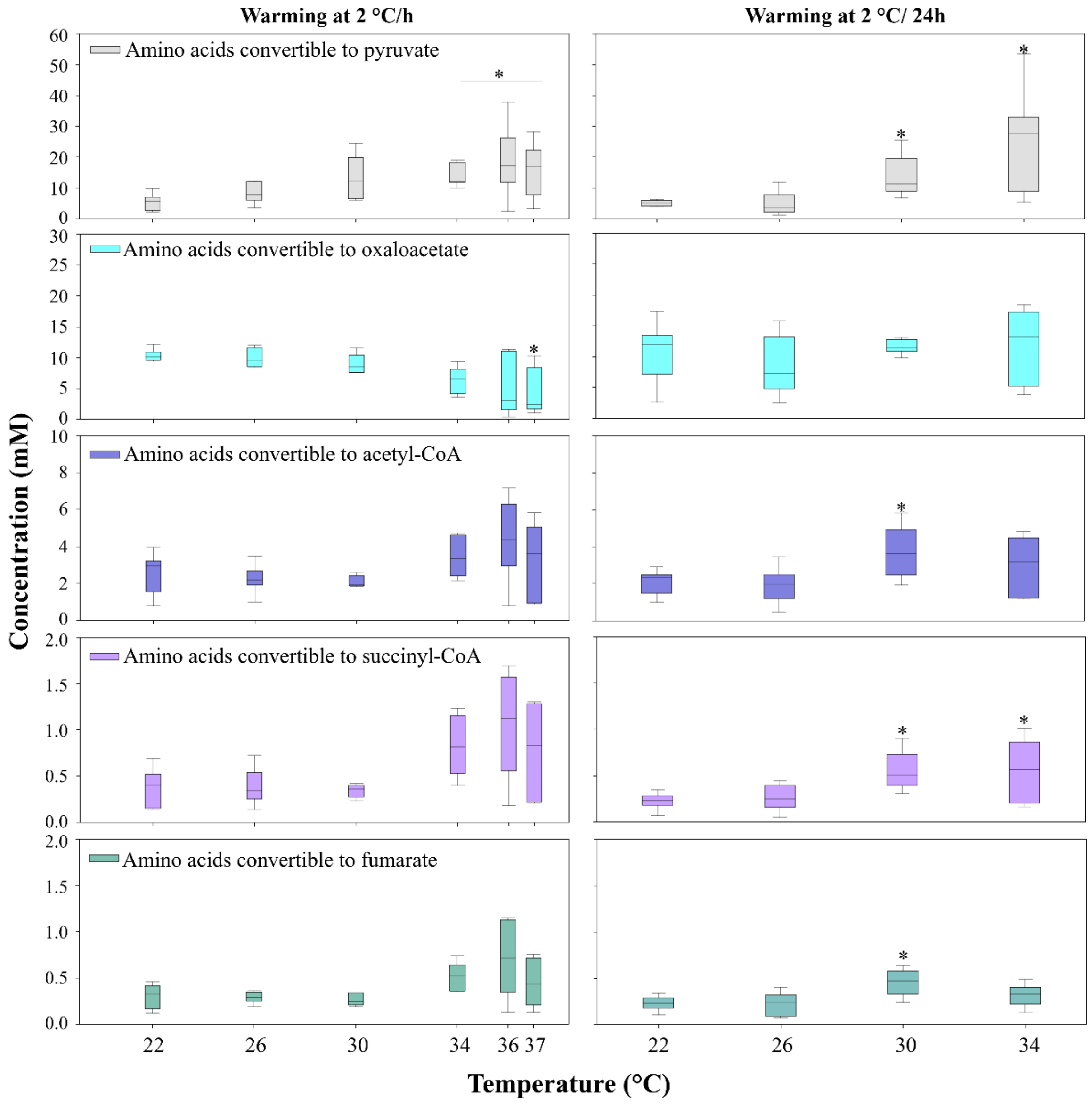

Fig. S2. Cardiomyocyte amino acids that enter or exit the TCA cycle generally increase with warming, except those convertible to oxaloacetate. Rapid and gradual warming shown in left and right columns, respectively; correlations between temperature and concentration were different between warming rates for amino acids convertible to pyruvate and for those convertible to oxaloacetate (see text). Concentrations of amino acids are summed in groups according to Table 1 in Owen et al. (2002; also see methods). Asterisks show significant differences from concentration at 22 °C tested by one-way ANOVA with Holm-Sidak correction for multiple comparisons.

Journal of Experimental Biology • Supplementary information

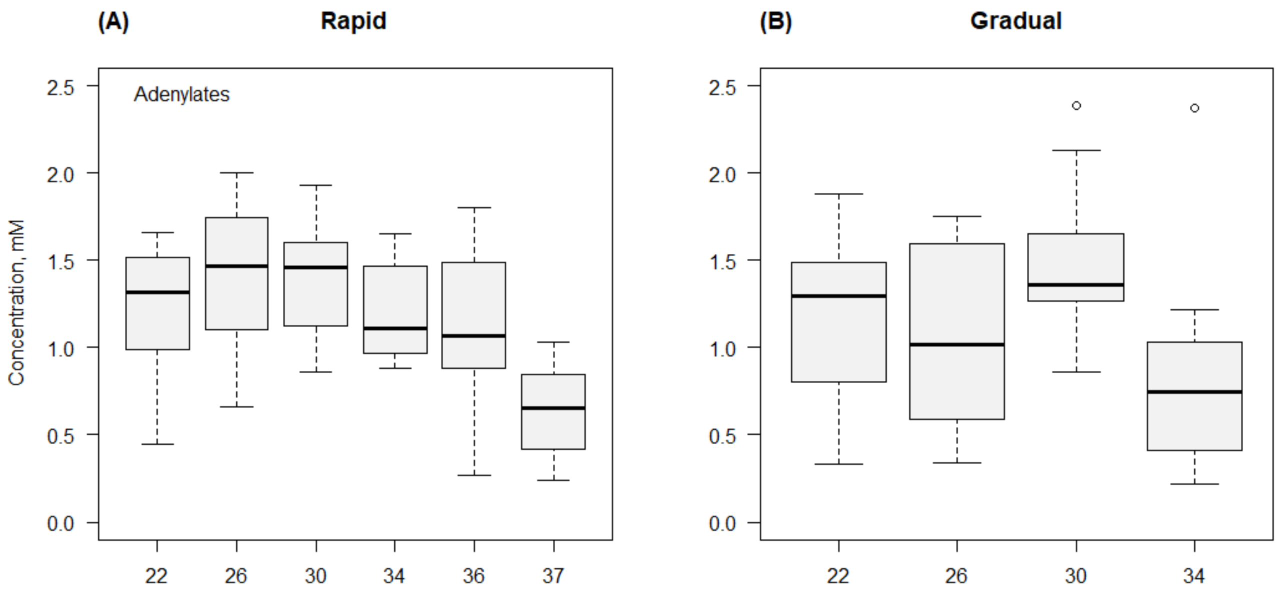

Fig. S3. Concentration changes of adenylates with warming at different rates. Note how final temperatures recorded in each panel, which just precede the the respective LT50s, are lowest in adenylates. Box plots show the median (bold line), interquartile range (box; IQR), and whiskers cover observations within 1.5 IQR.

Table S1. Summary of water chemistry parameters during rapid warming (2°C/h) and gradual warming exposures (2°C/24h). Temperature (T_{nom} = target temperature; T_{mean} = measured temperature in systems; °C), salinity (PSU), PO_2 (kPa), PCO_2 (µatm), $pH_{Freescale}$, and the total alkalinity (µmol/kg SW) were determined in water samples collected either at start and end of each exposure (under rapid warming) or throughout the exposures at each respective target temperature (gradual warming). Data are presented as means ± SD (each replicate ramp contains water chemistry data of three to five individual tank measurements).

	Temp (°C)			Salinity (PSU)	
	2°C/h	2°C/24h		2°C/h	2°C/24h
T_{nom}	Mean ± SD	Mean ± SD	T_{nom}	Mean ± SD	Mean ± SD
**22**	22.00 ± 0.09	22.04 ± 0.10	**22**	32.7 ± 1.3	33.3 ± 0.5
**24**	23.95 ± 0.07	24.03 ± 0.09	**24**		33.3 ± 0.5
**26**	25.93 ± 0.10	26.02 ± 0.11	**26**		33.2 ± 0.6
**28**	27.91 ± 0.08	28.05 ± 0.12	**28**		33.2 ± 0.8
**30**	30.00 ± 0.09	30.06 ± 0.16	**30**		32.8 ± 0.5
**32**	31.98 ± 0.10	32.00 ± 0.08	**32**		33.1 ± 0.5
**34**	33.99 ± 0.06	34.12 ± 0.07	**34**		33.3 ± 0.4
**36**	35.92 ±0.05	35.97 ± 0.07	**36**		33.5 ± 0.5
**38**	37.95 ± 0.07	-	**38**	32.6 ± 1.3	-

	PO_2 (kPa)			PCO_2 (µatm)	
	2°C/h	2°C/24h		2°C/h	2°C/24h
T_{nom}	Mean ± SD	Mean ± SD	T_{nom}	Mean ± SD	mean
**22**	21.5 ± 0.2	21.4 ± 0.4	**22**	450 ± 40	450 ± 40
**24**	21.4 ± 0.2	21.1 ± 0.2	**24**		458 ± 37
**26**	21.2 ± 0.2	20.9 ± 0.2	**26**		447 ± 37
**28**	20.9 ± 0.2	20.7 ± 0.2	**28**		453 ± 41
**30**	20.8 ± 0.2	20.3 ± 0.2	**30**		467 ± 55
**32**	20.6 ± 0.2	20.3 ± 0.3	**32**		493 ± 40
**34**	20.3 ± 0.2	20.2 ± 0.3	**34**		483 ± 29
**36**	20.1 ± 0.2	20.0 ± 0.2	**36**		464 ± 56
**38**	19.9 ± 0.2	-	**38**	582 ± 104	-

	$pH_{Freescale}$			Total alkalinity (µmol/kg SW)	
	2°C/h	2°C/24h		2°C/h	2°C/24h
T_{nom}	Mean ± SD	mean	T_{nom}	Mean ± SD	Mean ± SD
**22**	8.151 ± 0.03	8.112 ± 0.029	**22**	2556 ± 86	2099 ± 34
**24**		8.114 ± 0.020	**24**		2187 ± 321
**26**		8.134 ± 0.047	**26**		2125 ± 294
**28**		8.109 ± 0.056	**28**		2043 ± 294
**30**		8.100 ± 0.068	**30**		2050 ± 268
**32**		8.077 ± 0.059	**32**		2124 ± 283
**34**		8.112 ± 0.063	**34**		2136 ± 278
**36**		8.137 ± 0.057	**36**		2113 ± 364
**38**	8.109 ± 0.067	-	**38**	2547 ± 72	-

Table S2. Details on GAMM models presented in Fig. 2A and C. edf = effective degress of freedom, indicative of how curvilinear the effect of temperature is on HB. Ref. df. = reference degrees of freedom.

(A) Rapid warming

**Component**	**Term**	**Estimate**	**S.E.**	**t-value**	**p-value**	
Parametric coefficients	(Intercept)	1.93	0.09	20.7	<0.0001	***
**Component**	**Term**	**edf**	**Ref. df.**	**F-value**	**p-value**	
Smooth terms	s(temperature, k = 7)	5.94	5.94	339.4	<0.0001	***

Adjusted R-squared = 0.571; Scale est. = 1, n = 2697

(B) Gradual warming

**Component**	**Term**	**Estimate**	**S.E.**	**t-value**	**p-value**	
Parametric coefficients	(Intercept)	3.30	0.04	80.6	<0.0001	***
**Component**	**Term**	**edf**	**Ref. df.**	**F-value**	**p-value**	
Smooth terms	s(temperature, k = 8)	6.59	6.59	137.9	<0.0001	***

Adjusted R-squared = 0.569; Scale est. = 1, n = 9850

Journal of Experimental Biology • Supplementary information

Table S3. Summary of identified cardiomyocytes metabolites of *O. edulis* with significant changes under acute warming of 2 °C/h or gradual of 2°C/ 24h. Differences were detected by the online platform Metaboanalyst using a one-way ANOVA followed by Tukey's HSD. Shown are the compound, f-value, p-value, -log10(p), false discovery rate (FDR) and Tukey's HSD.

(A) Fast warming (2°C/h)

Metabolite	F-value	p-value	-log(10)p	FDR	Tukey's HSD
Succinate	21.27	<0.001	9.36	<0.001	22°C **vs.** 30 °C, 34°C, 36°C, 37°C<br>26°C, 30°C **vs.** 34°C, 36°C, 37°C
Alanine	5.58	<0.001	3.22	0.008	22°C **vs.** 34°C, 36°C, 37°C
Adenylates	3.72	0.008	2.11	0.048	22°C, 26°C, 30°C, 34°C vs. 37°C
Aspartate	3.63	0.009	2.05	0.048	30°C **vs.** 36°C, 37°C
Tauropine	3.57	0.01	2.02	0.048	22°C, 26°C **vs.** 36°C
Tyrosine	3.57	0.01	2.02	0.048	26°C **vs.** 36°C

(B) Gradual warming (2°C/24h)

Metabolite	F-value	p-value	-log(10)p	FDR	Tukey's HSD
Glycine	22.87	<0.001	8.28	<0.001	22°C, 26°C **vs.** 30°C, 34°C
Isoleucine	8.68	<0.001	3.89	0.002	22°C **vs.** 30°C, 34 °C<br>26°C **vs.** 30°C
Succinate	8.02	<0.001	3.63	0.002	22°C **vs.** 30°C, 34°C
Alanine	7.84	<0.001	3.55	0.002	22°C, 26°C **vs.** 34°C
beta-Alanine	7.25	<0.001	3.32	0.003	22°C, 26°C **vs.** 34°C
Valine	6.94	<0.001	3.19	0.003	22°C **vs.** 30°C, 34 °C
Lysine	5.93	0.002	2.75	0.007	22°C vs. 30°C, 34 °C<br>26°C vs. 30°C
UDP-N-Acetylglucosamine	5.77	0.002	2.68	0.008	22°C **vs.** 30°C
Hypotaurine	5.67	0.002	2.64	0.008	26°C **vs.** 34°C
Phenylalanine	5.52	0.003	2.57	0.008	22°C **vs.** 30°C
Threonine	5.14	0.004	2.4	0.01	26°C **vs.** 30°C
Leucine	5.13	0.004	2.4	0.01	22°C, 26°C **vs.** 30°C

Journal of Experimental Biology • Supplementary information

Table S4. Metabolic pathways of cardiomyocytes from *O.edulis* exposed to (A) rapid (2°C/h) or (B) gradual warming (2°C/24h) with a -log10(p) change of > 2.5. Shown are pathways including the total number of compounds (cmpd), the number and names of the identified hits, the raw p-value, the -log10(p) change, Holm adjusted p-values (accounting for multiple comparisons), and false discovery rate (FDR). Metabolites written in bold indicate a significant change determined in the ANOVA.

(A) Comparison of pathways affected by ramps

22 °C	Pathway	Total Cmpd	Hits	Compounds identified in the pathway	Raw p	[-LOG(p)]	Holm adjust	FDR
				no changes in pathways with [-LOG(p)] ≥ 2.5				
**26 °C**								
				no changes in pathways with [-LOG(p)] ≥ 2.5				
**30 °C**								
				no changes in pathways with [-LOG(p)] ≥ 2.5				
**34 °C**								
Carbohydrate metabolism	Butanoate metabolism	15	2	**Succinate**, Glutamate	0.001	3.01	0.03	0.017
	Citrate cycle (TCA cycle)	20	2	**Succinate**, Fumarate	0.001	2.99	0.03	0.017
	Propanoate metabolism	23	2	**Succinate**, ß-Alanine	0.002	2.80	0.05	0.017

Journal of Experimental Biology • Supplementary information

(B) Rapid warming (2°C/h)

22 *vs.*26 °C	Pathway	Total Cmpd	Hits	Compounds identified in the pathway	Raw p	[-LOG(p)]	Holm adjust	FDR
				no changes in pathways with [-LOG(p)] ≥ 2.5				
**22 *vs.*30 °C**								
Carbohydrate metabolism	Propanoate metabolism	23	2	**Succinate**, ß-Alanine	0.0004	3.36	0.01	0.008
	Citrate cycle (TCA cycle)	20	2	**Succinate**, Fumarate	0.0005	3.34	0.01	0.008
	Butanoate metabolism	15	2	**Succinate**, Glutamate	0.0009	3.02	0.03	0.010
**22 *vs.*34 °C**								
Carbohydrate metabolism	Citrate cycle (TCA cycle)	20	2	**Succinate**, Fumarate	< 0.0001	4.89	<0.01	<0.001
	Butanoate metabolism	15	2	**Succinate**, Glutamate	< 0.0001	4.61	<0.01	<0.001
	Propanoate metabolism	23	2	**Succinate**, ß-Alanine	< 0.0001	4.52	<0.01	<0.001
Amino acid metabolism	Alanine, aspartate and glutamate metabolism	27	6	Apartate, Asparagine, Fumarate, **Alanine**, Glutamate, **Succinate**	< 0.0001	4.52	<0.01	<0.001
	Tyrosine metabolism	42	2	Tyrosine, Fumarate	0.001	3.02	0.02	0.006
**22 *vs.*36 °C**								
Carbohydrate metabolism	Propanoate metabolism	23	2	**Succinate**, ß-Alanine	< 0.0001	5.70	<0.01	<0.001
	Citrate cycle (TCA cycle)	20	2	**Succinate**, Fumarate	< 0.0001	5.69	<0.01	<0.001
	Butanoate metabolism	15	2	**Succinate**, Glutamate	< 0.0001	5.53	<0.01	<0.001
Amino acid metabolism	Alanine, aspartate and glutamate metabolism	27	6	Apartate, Asparagine, Fumarate, **Alanine**, Glutamate, **Succinate**	< 0.0001	5.08	<0.01	<0.001

Journal of Experimental Biology • Supplementary information

(C) Gradual warming (2°C/h)

22 *vs.*26 °C	Pathway	Total Cmpd	Hits	Compounds identified in the pathway	Raw p	[-LOG(p)]	Holm adjust	FDR
				no changes in pathways with [-LOG(p)] ≥ 2.5				
**22 *vs.*30 °C**								
Amino acid metabolism	Glycine, serine and threonine metabolism	33	4	Choline - Betaine - Threonine - **Glycine**	<0.001	4.32	0.002	<0.001
	Valine, leucine and isoleucine degradation	40	3	Leucine - **Isoleucine** - **Valine**	<0.001	4.06	0.003	<0.001
	Valine, leucine and isoleucine biosynthesis	8	4	Leucine - **Isoleucine** - **Valine** - Threonine	<0.001	3.89	0.004	<0.001
	Phenylalanine, tyrosine and tryptophan biosynthesis	4	2	**Phenylalanine** - Tyrosine	<0.001	3.07	0.022	0.003
	Phenylalanine metabolism	8	2	**Phenylalanine** - Tyrosine	<0.001	3.07	0.022	0.003
Metabolism of other amino acids	Glutathione metabolism	28	2	**Glycine** - Glutamate	<0.001	4.16	0.002	<0.001
Carbohydrate metabolism	Glyoxylate and dicarboxylate metabolism	32	3	**Glycine** - Acetic acid - Glutamate	<0.001	3.62	0.007	0.001
	Butanoate metabolism	15	2	**Succinate** - Glutamate	0.001	2.86	0.029	0.003
	Propanoate metabolism	23	2	**Succinate** - ß-Alanine	0.002	2.68	0.038	0.004
Metabolism of cofactors and vitamins	Pantothenate and CoA biosynthesis	18	2	**Valine** - ß-Alanine	<0.001	3.01	0.023	0.003
Lipid metabolism	Glycerophospholipid metabolism	38	3	Acetylcholine - Choline - Phosphocholine	0.002	2.63	0.040	0.005
Genetic Information Processing	Aminoacyl-tRNA biosynthesis	48	13	Asparagine - **Phenylalanine** - **Glycine** - Asparate - **Valine** - **Alanine** - Lysine - **Isoleucine** - Leucine - Threonine - Tryptophan - Tyrosine - Glutamate	<0.001	3.66	0.006	0.001
**22 *vs.*34 °C**								
Amino acid metabolism	Glycine, serine and threonine metabolism	33	4	Choline - Betaine - Threonine - **Glycine**	<0.001	4.35	0.001	<0.001
	Alanine, aspartate and glutamate metabolism	27	6	Aspartate - Asparagine - Fumarate - **Alanine** - Glutamate - **Succinate**	0.002	2.66	0.055	0.007

Journal of Experimental Biology • Supplementary information

Metabolism of other amino acids	Glutathione metabolism	28	2	**Glycine** - Glutamate	<0.001	4.84	0.001	<0.001
Carbohydrate metabolism	Glyoxylate and dicarboxylate metabolism	32	3	**Glycine** - Acetic acid - Glutamate	<0.001	3.94	0.003	<0.001
	Propanoate metabolism	23	2	**Succinate - ß-Alanine**	<0.001	3.32	0.014	0.003
	Citrate cycle (TCA cycle)	20	2	**Succinate** - Fumarate	<0.001	3.09	0.023	0.004
	Butanoate metabolism	15	2	**Succinate** - Glutamate	0.001	3.01	0.026	0.005
Metabolism of cofactors and vitamins	Pantothenate and CoA biosynthesis	18	2	**Valine - ß-Alanine**	0.002	2.62	0.058	0.007
Genetic Information Processing	Aminoacyl-tRNA biosynthesis	48	13	Asparagine - Phenylalanine - **Glycine** - Asparate - **Valine** - **Alanine** - Lysine - **Isoleucine** - Leucine - Threonine - Tryptophan - Tyrosine - Glutamate	0.002	2.62	0.058	0.007

Journal of Experimental Biology • Supplementary information

Table S5. Effect of increasing temperature, rate of warming, and their interaction on amino acid summed concentrations, grouped according to their catabolic precursors. The default for rate of warming is the gradual warming "ramp". Statistics describe linear regressions, with significant interactions showing that the effect of temperature (slope) depends on the ramp.

	Coefficient	Df	Sum Sq	Mean Sq	F value	P	
(A) Pyruvate grouping							
(Intercept)	-12.42						
Temperature	0.81	1	2560.37	2560.37	40.23	0.000	***
Ramp	-26.33	1	192.07	192.07	3.02	0.086	.
Temperature *Ramp	1.00	1	461.30	461.30	7.25	0.009	**
Residuals		79	5028.23	63.65	NA	NA	
(B) Oxalocacetate grouping							
(Intercept)	19.91						
Temperature	-0.40	1	111.49	111.49	7.83	0.006	**
Ramp	-14.14	1	126.69	126.69	8.90	0.004	**
Temperature *Ramp	0.58	1	166.74	166.74	11.72	0.001	**
Residuals		76	1081.49	14.23	NA	NA	
(C) Acetyl-CoA grouping							
(Intercept)	0.00						
Temperature	0.10	1	26.76	26.76	14.86	0.000	***
Ramp	-0.61	1	0.12	0.12	0.07	0.797	
Temperature *Ramp	0.02	1	0.17	0.17	0.09	0.759	
Residuals		79	142.25	1.80	NA	NA	
(D) Succinyl-CoA grouping							
(Intercept)	-0.64						
Temperature	0.04	1	3.80	3.80	44.10	0.000	***
Ramp	0.12	1	0.31	0.31	3.56	0.063	.
Temperature *Ramp	-0.01	1	0.04	0.04	0.44	0.511	
Residuals		76	6.54	0.09	NA	NA	
(E) Fumarate grouping							
(Intercept)	-0.19						
Temperature	0.02	1	0.74	0.74	22.60	0.000	***
Ramp	0.16	1	0.09	0.09	2.67	0.106	
Temperature *Ramp	-0.01	1	0.03	0.03	0.93	0.337	
Residuals		77	2.52	0.03	NA	NA	

Journal of Experimental Biology • Supplementary information
